# Bmi‐1 high‐expressing cells enrich cardiac stem/progenitor cells and respond to heart injury

**DOI:** 10.1111/jcmm.13889

**Published:** 2018-11-05

**Authors:** Yuewang Song, Mengmeng Zhao, Yuan Xie, Tingfang Zhu, Wenbin Liang, Baiming Sun, Weixin Liu, Liqun Wu, Guoping Lu, Tao‐Sheng Li, Tong Yin, Yucai Xie

**Affiliations:** ^1^ Department of Cardiology Rui Jin Hospital Shanghai Jiao Tong University School of Medicine Shanghai China; ^2^ Bengbu Medical School Anhui Province China; ^3^ University of California, Santa Barbara Santa Barbara California; ^4^ Department of Cellular and Molecular Medicine University of Ottawa Ottawa ON Canada; ^5^ Cedars‐Sinai Heart Institute Los Angeles California; ^6^ Department of Stem Cell Biology Nagasaki University Graduate School of Biomedical Sciences Nagasaki Japan; ^7^ The National Research Center for Translational Medicine Rui Jin Hospital Shanghai Jiao Tong University School of Medicine Shanghai China

**Keywords:** Bmi‐1, infarction, stem/progenitor cell

## Abstract

Bmi‐1 gene is well recognized as an oncogene, but has been recently demonstrated to play a role in the self‐renewal of tissue‐specific stem cells. By using Bmi‐1^GFP^ ^/+^ mice, we investigated the role of Bmi‐1 in cardiac stem/progenitor cells and myocardial repair. RT‐PCR and flow cytometry analysis indicated that the expression of Bmi‐1 was significantly higher in cardiac side population than the main population from CD45^−^Ter119^−^ CD31^−^ heart cells. More Sca‐1^+^ cardiac stem/progenitor cells were found in Bmi‐1 GFP ^hi^ subpopulation, and these Bmi‐1 GFP ^hi^ heart cells showed the potential of differentiation into SMM ^+^ smooth muscle‐like cells and TnT^+^ cardiomyocyte‐like cells in vitro. The silencing of Bmi‐1 significantly inhibited the proliferation and differentiation of heart cells. Otherwise, myocardial infarction induced a significantly increase (2.7‐folds) of Bmi‐1 GFP ^hi^ population, mainly within the infarction and border zones. These preliminary data suggest that Bmi‐1^hi^ heart cells are enriched in cardiac stem/progenitor cells and may play a role in myocardial repair.

## 1 INTRODUCTION

Polycomb complex protein Bmi‐1 is encoded by the *BMI1* gene. A number of previous studies have demonstrated the roles of Bmi‐1 in the development and progression of various types of malignant tumours,^1^ such as leukaemia,^2^, ^3^ colorectal cancer,^4^ and medulloblastomas.^5^ These studies have found that down‐regulation of Bmi‐1 in cancer stem cells suppresses tumour growth.^3^, ^6^, ^7^ Beyond its role as an oncogene, up‐regulation of Bmi‐1 in various tissue‐specific stem cells,^8^, ^9^, ^10^ such as hematopoietic stem cells (HSC),^2^, ^3^, ^8^ intestinal stem cells,^11^ and epithelial stem cells in the pancreatic, prostate, lung, and others,^12^, ^13^, ^14^, ^15^, ^16^, ^17^, ^18^ has been demonstrated to play essential roles in the self‐renewal and the maintenance of stemness. Reduced expression of Bmi‐1 has also recently been found to enhance the beating of cardiomyocytes (CM) induced from neonatal and adult mouse fibroblasts by directly reprogramming.^19^ However, little has been known about Bmi‐1 expression in cardiac stem/progenitor cells.

Actually, the identity, origin and physiological role of endogenous cardiac stem/progenitor cells in adult mammals are still debated. For a long time, adult mammalian heart was thought to be a terminally differentiated organ. However, considerable evidence has shown the low turnover rate of CM.^20^, ^21^ There are at least two possible resources for the new born CM: preexisting CM^22^, ^23^ or cardiac stem/progenitor cells.^24^, ^25^, ^26^, ^27^ By now, different markers and methods have been applied for the identification and expansion of resident cardiac stem/progenitor cells, such as the c‐kit‐positive cells,^26^ Sca‐1‐positive cells,^27^ cardiac side population (SP),^24^ and cardiosphere derived cells.^28^ Using an inducible genetic labelling approach, we have recently defined cardioblasts, the small non‐myocyte cells express cardiac transcription factors and sarcomeric proteins and form mature CM in vivo after transplantation.^25^ Endogenous cardioblasts are rarely evident in the normal adult mouse heart, but will be significantly activated after myocardial infarction. The cardioblasts do not arise from haematogenous seeding, CM dedifferentiation, or mere expansion of a preformed progenitor pool.^25^

In this study, we investigated the potential role of Bmi‐1 on cardiac stem/progenitor cells by using Bmi‐1‐GFP‐knock‐in mice, in which GFP was expressed under the endogenous transcriptional regulatory elements of the Bmi‐1 gene, and the levels of Bmi‐1 expression in cells could be quantified by GFP fluorescence.^3^ We found that the subpopulations of cells with high expression of Bmi‐1 in heart tissue enriched in SP and Sca‐1‐positive cardiac stem/progenitor cells, and showed a significantly increase in number in response to myocardial infarction.

## 2 MATERIALS AND METHODS

### 2.1 Animals and genotyping

The procedures for all animal experiments were approved by the Animal Care and Use Committee of the Shanghai Ruijin Hospital, Shanghai Jiaotong University School of Medicine, China and the Cedars‐Sinai Medical Center, Los Angeles, CA, USA. All methods were performed in accordance with the relevant guidelines and regulations. Bmi‐1^GFP/+^ mice from JAX Lab, originally generated by Dr. Weissman group in Stanford University were inbred in the animal centre of Shanghai Ruijin Hospital, Shanghai, China. Eight‐ to 12‐week‐old mice were used for experiments. Mice genotyping was verified by PCR of tail genomic DNA.^3^

### 2.2 Evaluation of SP cells in heart cells and bone marrow cells

Heart SP and main population (MP) were prepared as previously described with modification.^24^ Briefly, heart tissue of Bmi‐1^GFP/+^ mice was minced into about 1 mm^3^ pieces and digested with 0.1% collagenase B (Roche Molecular Biochemicals, Mannheim, Gemany) and 2.4 U/mL dispase II (Roche Molecular Biochemicals) at 37°C for 30 minutes. After passing through a 50 μm filter, the CM‐depleted heart cells was washed and suspended in Hanks’ balanced salt solution (HBSS) buffer with 2% foetal calf serum and 10 mmol/L HEPES. Bone marrow cells were obtained from the same Bmi‐1^GFP/+^ mice as previously described.^29^ Single cell suspensions were incubated with Hoechst 33342 (5 g/mL) (Sigma, Shanghai, China) at 37°C for 90 minutes in DMEM (Cellgro, New York, NY, USA) (2% foetal calf serum, 10 mmol/L HEPES) at a concentration of 10^6^ nucleated cells/mL and washed in cold HBSS before cell surface antigen staining.^24^ Cell surface antigen staining was performed at 4°C for 30 minutes using fluorochrome conjugated monoclonal rat antimouse antibodies reactive to Sca‐1, CD31, and CD45 (all from Pharmingen, Shanghai, China). Respective isotype controls (Pharmingen) were used as negative controls. 7AAD was added before fluorescence‐activated cell sorting to exclude dead cells. Gates were established by forward and side scatters to exclude cellular debris. Fluorescent compensation was performed with single labelled controls. Quantitative flow cytometric assays were performed with a Cyan flow cytometer with Summit software (Beckman Coulter, Shanghai, China). Data were analysed with flowjo software (Ashland, OR, USA).

### 2.3 Cell culture and immunofluorescence staining

Purified CD45^−^Ter119^−^CD31^−^ Bmi‐1 high‐expressing (Bmi‐1^hi^) cells from heart cells were cultured in 1% fibronectin‐coated dishes with Iscove's modified Dulbecco's medium supplemented with 10% FBS, 100 U/mL penicillin, and 100 μg/mL streptomycin at 37°C in humidified air containing 5% CO_2_. Differentiation of CD45^−^Ter119^−^CD31^−^ Bmi‐1^hi^ cells was directed by supplementation of media of Trichostatin A.^30^ After 3 weeks cultured, cells were fixed by 4% PFA.

Cell were stained with rabbit polyclonal antibodies against Bmi‐1 (abcam), mouse monoclonal antibodies against cardiac Troponin T (abcam) and SMMHC/myosin (smooth muscle myosin (SMM) heavy chain; Biomedical Technologies), and then followed by fluorescence‐labelled secondary antibodies (Invitrogen, Carlsbad, CA, USA). Images were observed by GE DeltaVision OMX or Leica TCS SP5 confocal microscopy.

### 2.4 RNAi and RT‐qPCR

Bmi‐1 and the negative control siRNA duplexes were transfected into primary non‐CM cells using riboFECT™ CP (RIBOBIO) according to the manufacturer's instructions, target sequence 5′‐GCAGAUUGGAUCGGAAAGUTT‐3′. The efficiency of the corresponding gene silencing was validated by measuring the levels of mRNA expression by real‐time reverse transcriptase polymerase chain reaction (RT‐qPCR). Differentiation of cells was also directed by supplementation of media with Trichostatin A.

RT‐qPCR analysis was performed with SYBR Green on an ABI 7700 real‐time PCR machine (Applied Biosystems, Shanghai, China) according to the manufacturer's instructions. The expression level of Bmi‐1 was normalized to internal control. Bmi‐1 primer sequence: forward, ATCCCCACTTAATGTGTGTCCT; reverse, CTTGCTGGTCTCCAAGTAACG.

### 2.5 Myocardial infarction model

To further investigate how the Bmi‐1 expression will be changed in response to heart injury, myocardial infarction was made in Bmi‐1^GFP/+^ mice (8‐12 weeks old) by permanent ligation of the left anterior descending coronary artery (LAD). Mice were randomly allocated into either LAD ligation or sham operation group by random number table, 9 mice/group in total. Mice were performed with tracheal intubation, tidal volume 0.7 mL, respiratory rate 120 breaths per minute. A left thoracotomy was performed through the fourth intercostal space. After removing the pericardium, LAD was ligated with 7‐0 silk suture under direct vision of surgical microscopy. Mice received a left thoracotomy alone were used for control. All mice survived after the successful surgical procedures, and no mouse died during the follow‐up period. Six mice from each group were euthanized 1 week after surgery, and the heart for evaluating the subpopulation of cells with high expression of Bmi‐1 by flow cytometry as described above. Another three mice from each group were euthanized 2 weeks after surgery, and excised hearts were snap‐frozen for histological analysis, and cryosections (10 μm) were stained following standard procedures. Images of histological staining were taken using Olympus microscope. All analyses were conducted by individuals blind to treatment allocation.

### 2.6 Statistical analysis

Data and statistical analysis were done by GraphPad Prism 6.0. Results are presented as mean ± SEM unless specified otherwise. Comparisons between any two groups were performed with two‐tailed unpaired Student's *t*‐test. Each experiment was performed at least three times and the differences were considered statistically significant when *P* < 0.05.

## 3 RESULTS

### 3.1 Bmi‐1 GFP^hi^ subpopulation enriched not only in HSC but also in cardiac stem/progenitor cells

**Figure 1:**
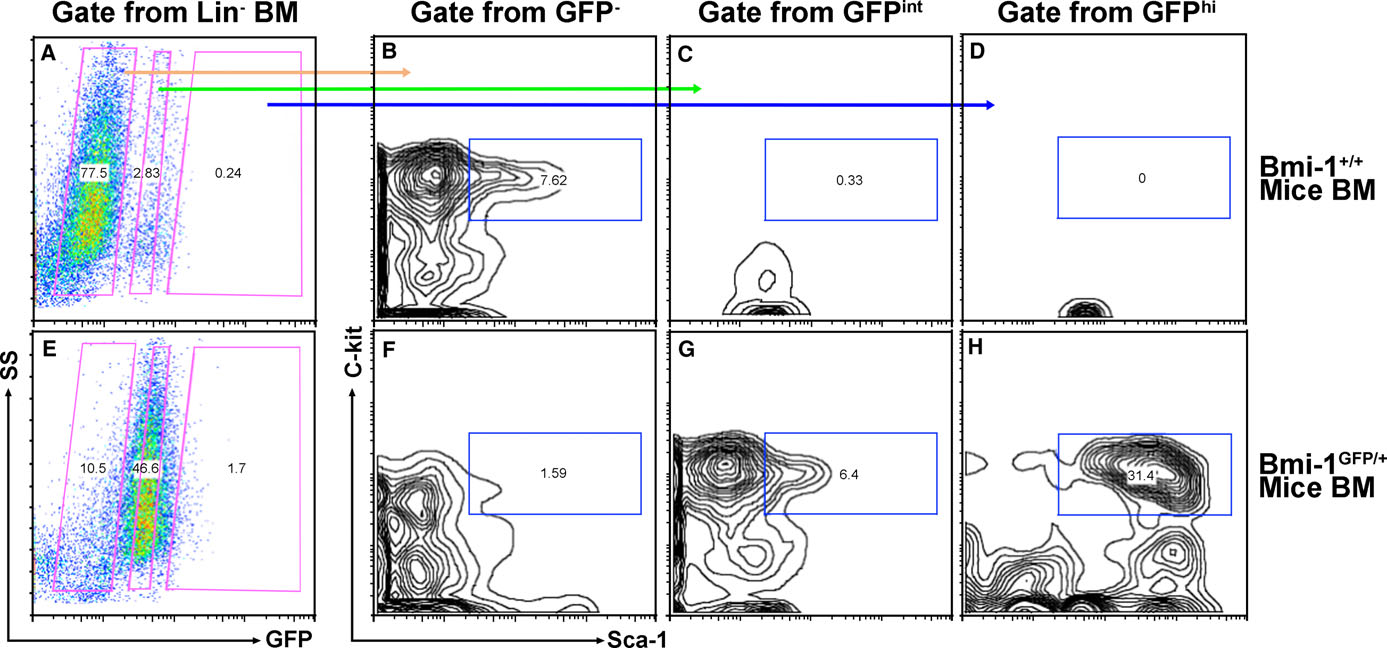
Hematopoietic stem cells were enriched in Bmi‐1 GFP ^hi^ population from bone marrow. Representative flow cytometry plots of bone marrow cells from Bmi‐1^+/+^ mice (A‐D) or Bmi‐1^GFP^ ^/+^ mice (E‐H). (A) and (E) were from Lin^−^ bone marrow population, then further separate into Bmi‐1 GFP‐negative, intermediate or high population (B‐D and F‐H) based on the expression of Sca‐1 and c‐kit to get lin^−^C‐kit^+^Sca‐1^+^ HSCs in different GFP expression population. BM: bone marrow

Based on the GFP fluorescence intensity, we divided the lineage‐negative (Lin^−^) bone marrow cells from Bmi‐1^GFP/+^ mice into GFP high (hi), intermediate (int), and negative (−) subpopulations (Figure 1). As congruent with previous report,^3^ HSC (Lin^−^c‐kit^+^Sca‐1^+^ HSCs) were dramatically enriched in the GFP^hi^ cells when compared to the GFP^int^ and GFP^−^ cells (Figure 1F‐H). Similarly, higher expression of GFP was also observed in the Lin^−^c‐kit^+^Sca‐1^+^ HSCs than the Lin^−^c‐kit^+^ progenitors or Lin^+^ matured cells from bone marrow of Bmi‐1^GFP/+^ mice (Figure S1).

**Figure 2:**
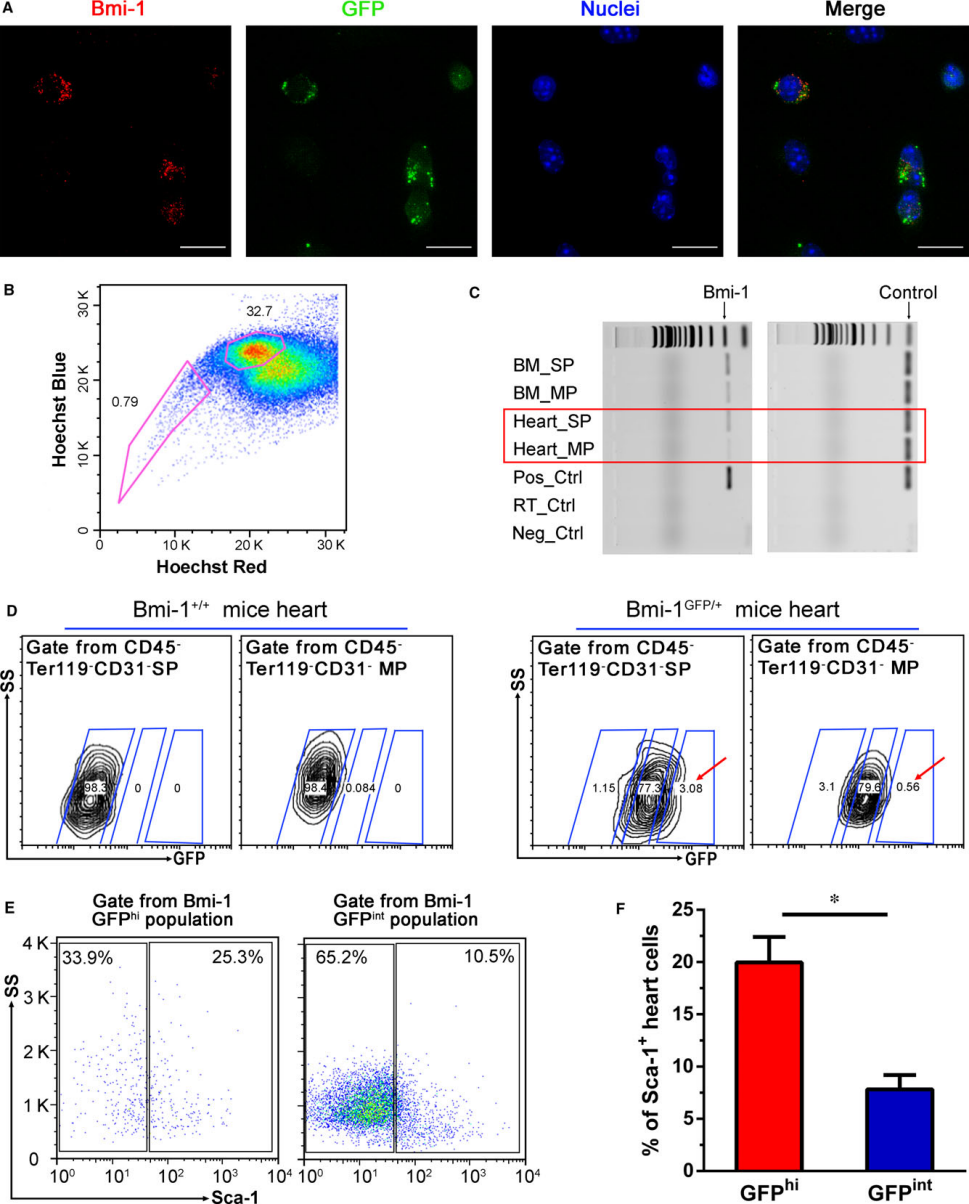
Relationship between Bmi‐1 GFP ^hi^ cells and cardiac stem/progenitor cells. (A) GE DeltaVision OMX images showed that the coincidence of GFP with Bmi‐1 expression. Scale bars, 15 μm. (B) Representative flow plot for cardiac side population (SP) and main population (MP) from CD45^−^Ter119^−^ CD31^−^ heart cells. (C) RT‐PCR indicated higher Bmi‐1 expression in cardiac SP than MP population. The grouping of gels was from the same gel. (D) Flow cytometry plots showed Bmi‐1 GFP ^hi^ heart cells were enriched in SP population (red arrow). Representative flow cytometry plots (E) and quantitative data (F) showed more Sca‐1^+^ cells in Bmi‐1 GFP ^hi^ than GFP ^int^ populations of heart cells. Sca‐1^+^ cells were gated from CD45^−^Ter119^−^ CD31^−^ heart cells. *P < 0.05

We next examined whether Bmi‐1 will be also highly expressed in cardiac stem/progenitor cells. Because almost all GFP^+^ cells were Bmi‐1^+^ and 96.69% ± 2.61% of Bmi‐1^+^ heart cells expressed GFP through immunofluorescent images (Figure 2A), we used GFP^+^ cells to represent Bmi‐1^+^ cells in flow cytometry assay. After negative deletion of matured hematopoietic cells and endothelial cells by using antibodies against CD45, Ter119, and CD31, we divided these CD45^−^Ter119^−^CD31^−^ heart cells into cardiac SP and MP as the previously stated^24^ (Figure 2B). We found that the Bmi‐1 expression was significantly higher in SP cells than in MP cells (Figure 2C). We also tried to gate CD45^−^Ter119^−^CD31^−^ SP cells by flow cytometry, and then measured the Bmi‐1 expression by fluorescence intensity. We could observe more Bmi‐1 GFP^hi^ cells in SP population than MP population (3.73 ± 0.76% vs 1.10 ± 0.38%, *P* < 0.05; Figure 2D). Sca‐1 was known as one of the common marker for stem/progenitor cells. Our results also showed that Sca‐1^+^ cardiac stem/progenitor cells were enriched in Bmi‐1 GFP^hi^ population from CD45^−^Ter119^−^CD31^−^ heart cells (Figure 2E). The percentage of Sca‐1^+^ cells gated from Bmi‐1 GFP^hi^ heart cells was almost 2.5‐folds higher than that of from Bmi‐1 GFP^int^ population (19.98 ± 4.88% vs. 7.82 ± 2.77%, *P* < 0.05; Figure 2F).

So, our data from BM and heart cells indicated that Bmi‐1 GFP^hi^ subpopulation enriched not only in HSC but also in cardiac stem/progenitor cells.

### 3.2 Bmi‐1 GFP^hi^ heart cells could differentiate into SMM^+^ smooth muscle‐like cells and TnT^+^ CM‐like cells in vitro

**Figure 3:**
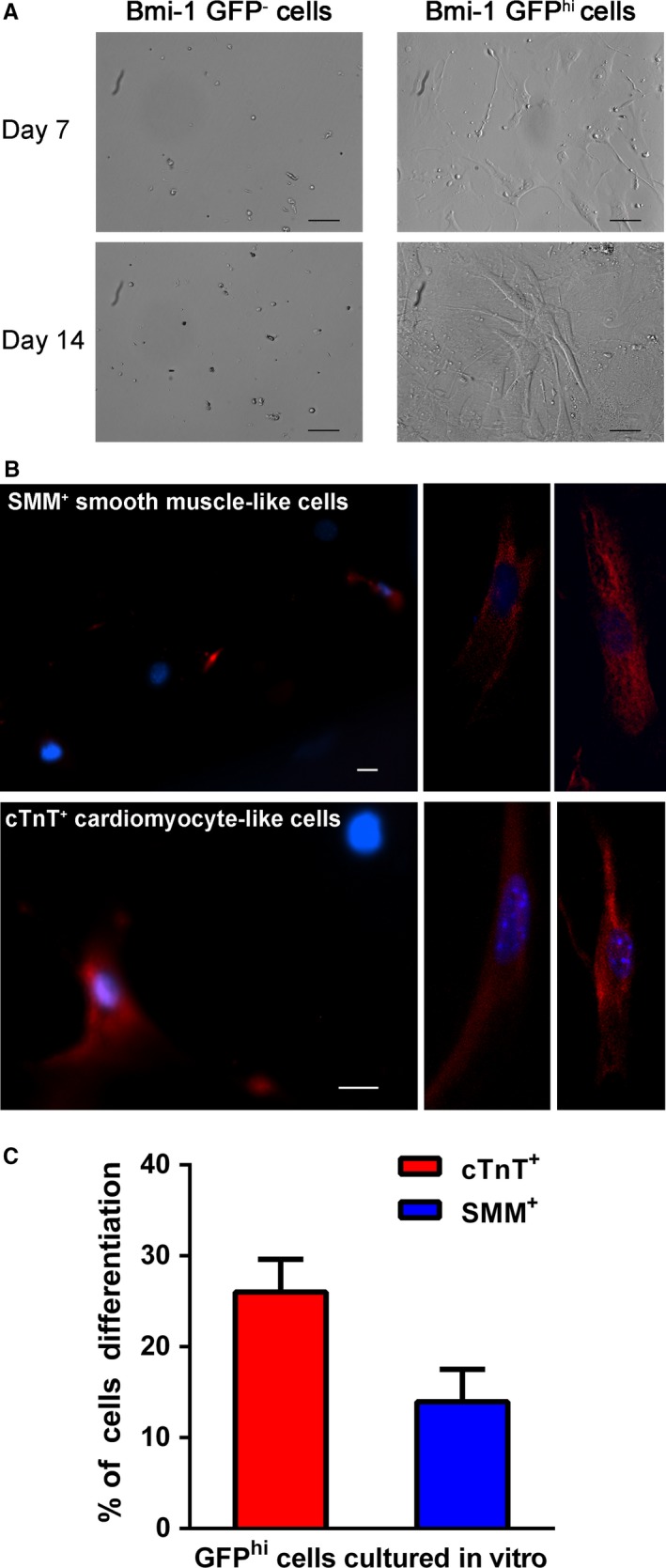
In vitro growth and differentiation of Bmi‐1 GFP ^hi^ heart cells sorted from Bmi‐1^GFP^ ^/+^ mice. (A) Bmi‐1 GFP ^hi^ cells grew well at day 7 and day 14, but no cells grew from the Bmi‐1 GFP ^−^ cells. Scale bars, 50 μm. Representative confocal images (B) and quantitative data (C) indicated the differentiation of Bmi‐1 GFP ^hi^ cells into SMM ^+^ smooth muscle‐like cells and TnT^+^ cardiomyocyte‐like cells in vitro. Scale bars, 20 μm

We also purified CD45^−^Ter119^−^D31^−^ Bmi‐1 GFP^hi^ and GFP^−^ subpopulations from Bmi‐1^GFP/+^ mice heart, and then evaluated their potency of myocardial differentiation in vitro (Figure 3). These Bmi‐1 GFP^hi^ cells grew well after 7 and 14 days of culture**,** but cells were rarely grown from the Bmi‐1 GFP^−^ cells (Figure 3A). Immunostaining showed that some cells grown from Bmi‐1 GFP^hi^ cells were positively expressed with SMM heavy chain (12.96% ± 2.70%) and cardiac Troponin T (TnT) (26.03% ± 3.58%), suggesting the differentiation into smooth muscle‐like cells and CM‐like cells (Figure 3B,C).

### 3.3 Bmi‐1 knockdown inhibited the proliferation and myocardial differentiation of non‐CM cells

**Figure 4:**
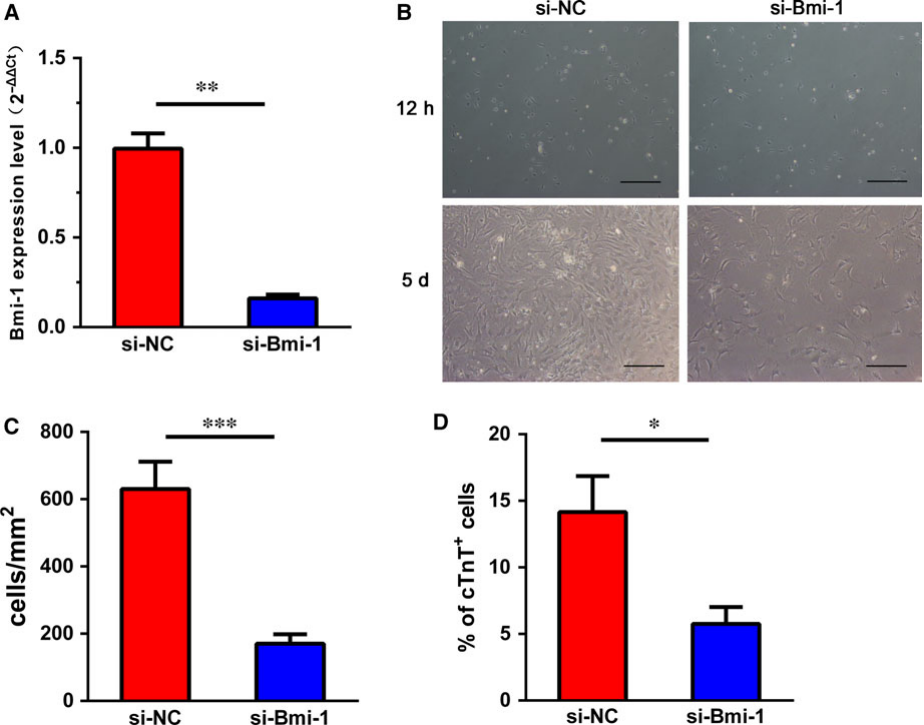
Bmi‐1 knockdown inhibited the proliferation and differentiation of non‐cardiomyocytes. (A) Bmi‐1 mRNAs were significantly reduced by siRNA transfection. Representative phase contrast images (B) and quantitative data (C) showed the growth of cells at 5 days after TSA treatments. Scale bars, 200 μm. (D) The proportion of cTnT ^+^ cardiomyocyte‐like cells was at 5 days after TSA treatments. NC: negative control. *P < 0.05; **P < 0.005; ***P < 0.001

To confirm the role of Bmi‐1 on the proliferation and differentiation, we tried to silence the expression of Bmi‐1 in non‐CM cells by siRNA (Figure 4). The efficiency of knockdown was showed in Figure 4A. The silencing of Bmi‐1 significantly inhibited the proliferation of non‐CM cells (170.5 ± 27.54 vs 630.5 ± 80.87 cells/mm^2^, *P* < 0.001, Figure 4B,C) and differentiation of cTnT^+^ CM‐like cells (5.76% ± 1.27% vs 14.16% ± 2.70%, *P* < 0.05, Figure 4D) at 5 days after TSA treatment.

### 3.4 Bmi‐1 GFP^hi^ population increased in response to myocardial infarction

**Figure 5:**
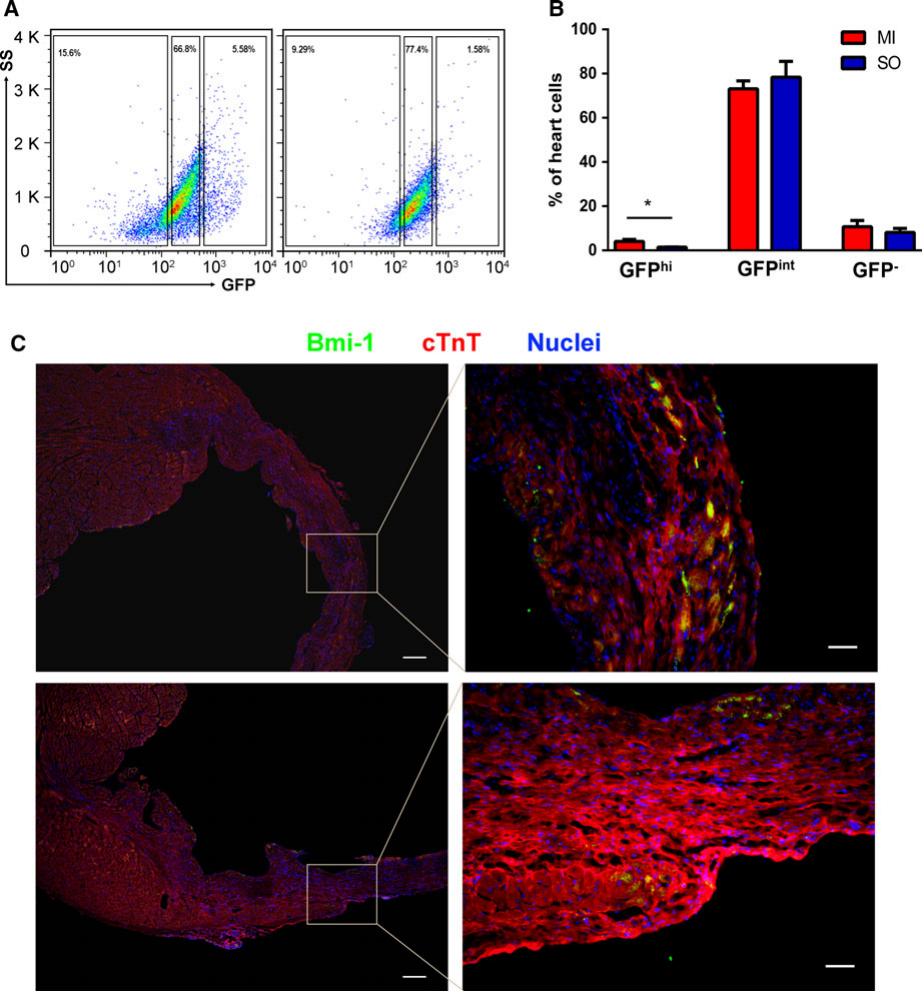
Bmi‐1 GFP ^hi^ population increased in myocardial infarction. Representative flow cytometry plots (A) and quantitative data (B) on GFP ^hi^, GFP ^int^, and GFP ^‐^ subpopulations in heart cells of mice 1 week after myocardial infarction (MI) and sham operation (SO). (C) Immunofluorescence staining indicated some clusters of Bmi‐1^hi^ cells within the infarction (Upper) and border (Lower) zones at 2 weeks after myocardial infarction. Scale bars, 200 μm (left), 50 μm (right). *P < 0.05

To further evaluate the role of Bmi‐1 expression in myocardial repair, we analysed the change of Bmi‐1 GFP^hi^ heart cells in Bmi‐1^GFP/+^ mice after LAD ligation. Interestingly, CD45^−^Ter119^−^CD31^−^ Bmi‐1 GFP^hi^ population was significantly increased in the infarcted heart when compared to the control heart received sham operation (4.04 ± 1.55% vs 1.47 ± 0.12%, *P *< 0.05). However, myocardial infarction did not induce significant changes in the GFP^int^ and GFP^−^ populations (Figure 5A,B). Immunofluorescence staining also clearly showed some clusters of Bmi‐1^hi^ cells within the infarction and border zones (Figure 5C). Moreover, many CM were observed around the clusters of Bmi‐1^hi^ cells. Although the absence of direct evidence, these results indirectly suggested that the heart cells expressed with high level of Bmi‐1 might be involved in myocardial repair after injury.

## 4 DISCUSSION

As congruent with a previous report,^3^ our data indicated the percentage of lin^−^c‐kit^+^Sca‐1^+^ HSC were much higher in Bmi‐1 GFP^hi^ population compared to GFP^int^ and GFP^−^ cells in Bmi‐1^GFP/+^ mice (Figure 1). On the other hand, Bmi‐1 GFP was highly expressed in lin^−^c‐kit^+^Sca‐1^+^ HSC when compared with Lin^−^c‐kit^+^ progenitors or Lin^+^ matured cells from bone marrow (Figure S1). Actually, the high level of Bmi‐1 expression has been demonstrated to be critical on the self‐renewal of HSC and the maintenance of hematopoietic function.^8^, ^31^

Although cardiac‐specific Bmi‐1 deletion during embryogenesis does not affect cardiogenesis,^32^ it has been reported that quercetin could minimize doxorubicin‐induced cardiotoxicity by modulating Bmi‐1 expression.^33^ There is very limited information on the role of Bmi‐1 expression in cardiac stem/progenitor cells. By using Bmi‐1‐GFP‐knock‐in mice,^3^ we found that the expression with high level of Bmi‐1 in heart cells enriched in cardiac stem/progenitor cells. The knockdown of Bmi‐1 significantly reduced the total number of non‐CM cells and the percentage of cTnT^+^ cells in vitro. Furthermore, in response to myocardial infarction, only the Bmi‐1 high‐expressing cells were increased about 2.7‐folds in the infarcted heart, mainly observed in the infarction and border zones, which suggests a potential role for myocardial repair after injury.

Agreed with our findings, Valiente‐Alandi I et al has crossed the Bmi‐1^CreER/+^ strain with Rosa26^YFP/+^ reporter mice to generate Bmi‐1^CreER/+^;Rosa26^YFP/+^ (Bmi‐1‐YFP) mice for cardiac lineage tracing, and demonstrated that Bmi‐1^+^ cells contribute to myocardial renewal^34^ and myocardial repair following acute injury.^35^ Furthermore, Bmi‐1 expression is associated with reactive oxygen species levels.^36^ But we made a more detailed distinction. In this study, we used Bmi‐1‐GFP‐ transgenic mice which enable us to distinguish the Bmi‐1 high‐expressing cells from the cells with intermediate level of Bmi‐1 expression. Interestingly, among the Bmi‐1‐positive cells, only these Bmi‐1 high‐expressing cells enriched in cardiac stem/progenitor cells and was increased after heart injury (Figures 2C,F and 5A).

Our study has several limitations. First, although heart cells with high level of Bmi‐1 expression enriched in cardiac stem/progenitor cells, these Bmi‐1^hi^ cells were partially expressed with common stem/progenitor cell makers of Sca‐1 and also partially included in the SP, suggested the heterogeneity of Bmi‐1^hi^ cells. Therefore, it is still asked to further rationalize the Bmi‐1 as a novel marker for the identification of cardiac stem/progenitor cells. Second, it is impossible for us to exactly quantify the Bmi‐1^hi^ cells in the whole heart because we collected these small size heart cells for analysis by removing the CM. Third, we do not know whether the subpopulations of small size heart cells with the expression of Bmi‐1 at intermediate and low levels are actually generated from these Bmi‐1^hi^ cells followed by a gradually reduction of Bmi‐1 expression during the differentiation process.^3^ Finally, it is hard for us to collect enough number of Bmi‐1^hi^ heart cells for in vivo implantation into a damaged heart. The role of Bmi‐1 is also required to investigate in vivo by using Bmi‐1 knockout animals. So, the role of Bmi‐1^hi^ heart cells for functional myocardial repair is still questionable.

In summary, Bmi‐1 was expressed higher in cardiac SP than MP from CD45^−^Ter119^−^CD31^−^ heart cells. More Sca‐1^+^ cells were found in Bmi‐1 GFP^hi^ population, and more Bmi‐1 GFP^hi^ cells in Sca‐1^+^ population. The CD45^−^Ter119^−^CD31^−^ Bmi‐1 GFP^hi^ cells from Bmi‐1^GFP/+^ mice could differentiate into SMM^+^ smooth muscle‐like cells and TnT^+^ CM‐like cells in vitro. The silencing of Bmi‐1 significantly inhibited the proliferation and differentiation of heart cells. Bmi‐1 GFP^hi^ population increased in heart of mice 1 week after infarction and some clusters of Bmi‐1^hi^ cells were observed within the infarction and border zones. Based on our data, heart cells with high Bmi‐1 expression seem to be enriched in cardiac stem/progenitor cells and possibly play a role during myocardial repair.

## CONFLICT OF INTEREST

The authors confirm that there are no conflicts of interest.

## AUTHOR CONTRIBUTION

Yuewang Song, Guoping Lu, Taosheng Li, Tong Yin, and Yucai Xie designed the study. Yuewang Song, Mengmeng Zhao, Yuan Xie, Tingfang Zhu, Baiming Sun, Weixin Liu, Liqun Wu, Tong Yin, and Yucai Xie performed the experiments. Yuewang Song, Wenbin Liang, Tingfang Zhu, Taosheng Li, Tong Yin, and Yucai Xie analysed the data. Yuan Xie, Taosheng Li, Tong Yin, and Yucai Xie wrote the manuscript. Yuewang Song, Mengmeng Zhao, Yuan Xie should be considered equal first authors.
