# Supplementary material for: Bmi‐1 high‐expressing cells enrich cardiac stem/progenitor cells and respond to heart injury
Source: J Cell Mol Med. 2018 Nov 5;23(1):104–11. doi: 10.1111/jcmm.13889 (PMC6307799; doi:10.1111/jcmm.13889)

**Bmi-1 high-expressing cells enrich cardiac stem/progenitor cells and respond to heart injury**

Yuewang Song ^a #^, Mengmeng Zhao ^a, b #^, Yuan Xie ^a,c #^, Tingfang Zhu ^a^，Wenbin Liang ^d^, Baiming Sun ^d^, Weixin Liu ^d^, Liqun Wu ^a^, Guoping Lu ^a^, Taosheng Li ^e *^, Tong Yin ^f *^ & Yucai Xie ^a, d *^

^a^ Department of Cardiology, Rui Jin Hospital, Shanghai Jiao Tong University School of Medicine, Shanghai, China

^b^ Bengbu Medical School, Anhui Province, China

^c^ University of California Santa Barbara, California, USA

^d^ Cedars-Sinai Heart Institute, Los Angeles, California, USA

^e^ Department of Stem Cell Biology, Nagasaki University Graduate School of Biomedical Sciences, Nagasaki, Japan

^f^ The National Research Center for Translational Medicine, Shanghai Jiao Tong University School of Medicine, Shanghai, China

# These authors contributed equally to this work.

* Correspondence to: Yucai Xie (197, Rui Jin Er Road, Shanghai, Tel: +86 21 64370045, E-mail: [drxieyucai@163.com](mailto:drxieyucai@163.com)) or Tong Yin (197, Rui Jin Er Road, Shanghai, Tel: +86 21 64370045, E-mail: [yintong0101@163.com](mailto:yintong0101@163.com)) or Taosheng Li (1-12-4 Sakamoto, Nagasaki 852-8523, Japan, Tel: 81-95-819-7099, E-mail: [litaoshe@nagasaki-u.ac.jp](mailto:litaoshe@nagasaki-u.ac.jp))

**Supplementary figure 1. Percentage of Bmi-1 GFP^+^ cells is the highest in hematopoietic stem cells.** Representative flow cytometry plots for HSCs **(A-C),** Lin-C-kit^+^ progenitors **(D-F)** or Lin^+^ mature cells **(G-I)** gated from bone marrow of Bmi-1^+/+^ mice **(A, D, G)** or Bmi-1^GFP/+^ mice **(B, E, H).** Bmi-1 GFP was expressed at the highest levels in HSC cells **(C)** compared with other populations. BM: bone marrow.


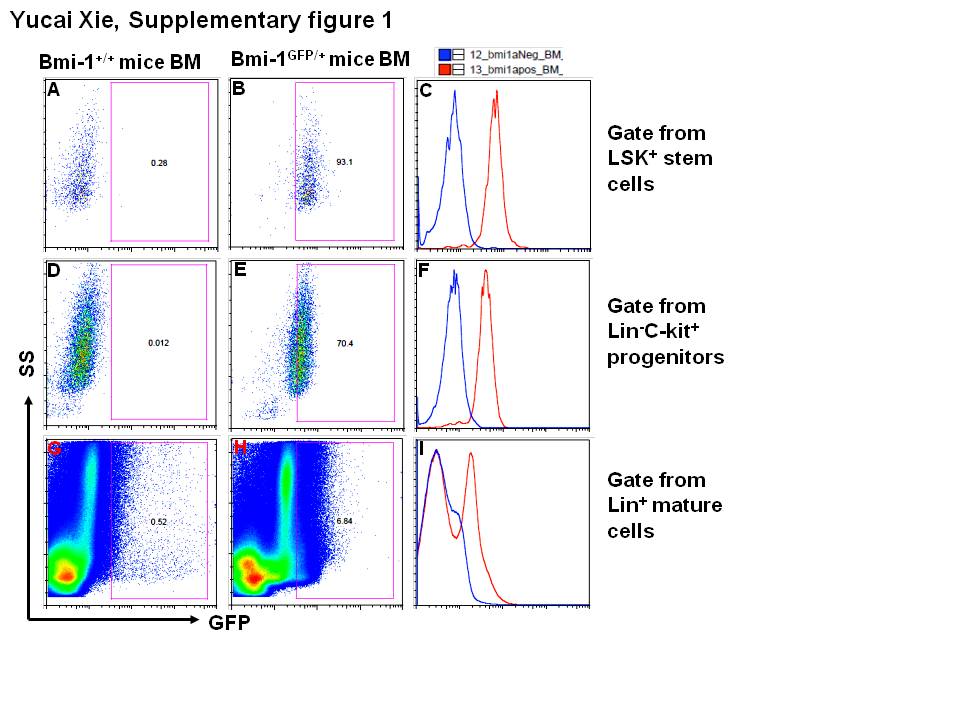

Supplement: Supplementary file 1 [file JCMM-23-104-s001.doc]
